# Campylobacteriosis associated with the consumption of unpasteurised milk: findings from a sentinel surveillance site

**DOI:** 10.1017/S0950268819002292

**Published:** 2020-02-04

**Authors:** G. Davys, J. C. Marshall, A. Fayaz, R. P. Weir, J. Benschop

**Affiliations:** 1 School of Veterinary, Animal and Biomedical Sciences, Massey University, Palmerston North, New Zealand; 2 School of Fundamental Sciences, Massey University, Palmerston North, New Zealand; 3 Molecular Epidemiology and Public Health Laboratory, School of Veterinary, Animal and Biomedical Sciences, Massey University, Palmerston North, New Zealand; 4 MidCentral Public Health Services, MidCentral District Health Board, Palmerston North, New Zealand

**Keywords:** Campylobacter, food-borne infections, notifiable infectious diseases, public health, raw milk collection

## Abstract

Campylobacteriosis is the most common notifiable disease in New Zealand. While the risk of campylobacteriosis has been found to be strongly associated with the consumption of undercooked poultry, other risk factors include rainwater-sourced drinking water, contact with animals and consumption of raw dairy products. Despite this, there has been little investigation of raw milk as a risk factor for campylobacteriosis. Recent increases in demand for untreated or ‘raw’ milk have also raised concerns that this exposure may become a more important source of disease in the future. This study describes the cases of notified campylobacteriosis from a sentinel surveillance site. Previously collected data from notified cases of raw milk-associated campylobacteriosis were examined and compared with campylobacteriosis cases who did not report raw milk consumption. Raw milk campylobacteriosis cases differed from non-raw milk cases on comparison of age and occupation demographics, with raw milk cases more likely to be younger and categorised as children or students for occupation. Raw milk cases were more likely to be associated with outbreaks than non-raw milk cases. Study-suggested motivations for raw milk consumption (health reasons, natural product, produced on farm, inexpensive or to support locals) were not strongly supported by cases. More information about the raw milk consumption habits of New Zealanders would be helpful to better understand the risks of this disease, especially with respect to increased disease risk observed in younger people. Further discussion with raw milk consumers around their motivations may also be useful to find common ground between public health concerns and consumer preferences as efforts continue to manage this ongoing public health issue.

## Introduction

Although the milk of healthy cows usually contains few bacteria at the source, it can easily become contaminated during collection via contact with pathogenic bacteria from the skin and teats of the cow, the interior surfaces of the milking machine and the hands of those associated with the milking process [1]. Once contaminated, milk is an ideal culture medium and can support rapid microbial growth, increasing the risk of disease [2,3]. Rates of milk-associated disease fell precipitously following the introduction of pasteurisation in the 1940s and are comparatively very low in the current era [4]. Despite this, some people still currently choose to consume raw milk and, for this group, milk-associated disease remains a risk. *Campylobacter* spp. are the most common pathogens reported in association with raw milk-related disease outbreaks in New Zealand, a pattern that is also seen internationally [5,6].

In New Zealand, raw milk can be legally purchased directly from registered farms and is also commonly consumed by farm residents and employees [5]. A survey of New Zealand dairy farmers found that 65% reported consuming raw milk [7]. In contrast, raw milk consumers are believed to make up only a small proportion of the general population. However, demand for raw milk in New Zealand has increased in recent years, in line with a rise in interest in natural and locally produced food products [8]. Many consumers also report perceived health benefits in association with raw milk consumption [7]. In the USA, the number of notified raw milk-associated disease outbreaks has likewise increased, with nearly twice the number of outbreaks reported in 2007–2012 compared to 1993–2006 [6,9,10].

There has been recent consultation around raw milk legislation in New Zealand [8] with pressure from raw milk advocates to facilitate easier access to this product. Therefore, the analysis of notified raw milk-associated disease is timely. The current study aims to describe the demographics of raw milk usage and temporal distribution of raw milk-associated campylobacteriosis cases from a sentinel site and compare them to notified campylobacteriosis cases from the same region without raw milk exposure.

## Materials and methods

### Study area

**Fig. 1.**
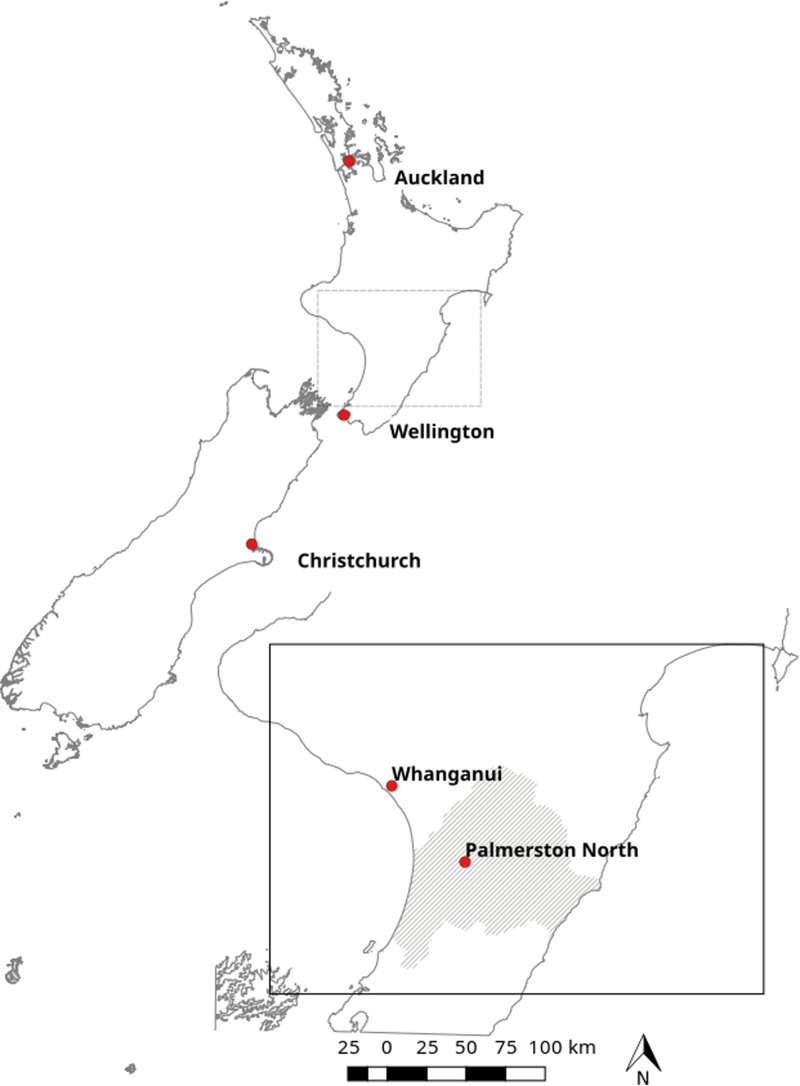
Map of New Zealand with major cities labelled. The area administered by the MidCentral District Health Board (shaded) is shown as inset.

This was an observational, retrospective study of data collected in collaboration between MidCentral District Health Board (DHB) and Massey University's Molecular Epidemiology and Public Health Laboratory (^m^EpiLab) during the period 2012–2017 inclusive. The study area was located in the North Island of New Zealand, with a population of approximately 170 000 people [11]. The majority of the study population lived in urban areas; however, the region contained a sizeable rural population [12]. The MidCentral DHB region is shown, in the context of greater New Zealand, in Figure 1.

### Data collection

Cases of campylobacteriosis occurring within the MidCentral DHB area had been captured previously as part of a sentinel surveillance programme in the area [12]. Additional funding, provided to the sentinel site from 2005 to 2017 (inclusive), had allowed the local Public Health Unit to enhance routine, passive disease surveillance with active case investigation and molecular epidemiology during this period [13,14]. Following notification, cases were investigated, using telephone or in-person interview, by members of the local Public Health Unit and the information obtained was added to New Zealand's national notifiable disease surveillance database ‘EpiSurv’. Reported consumption of raw milk during the initial interview prompted further investigation using a specific raw milk questionnaire. This additional investigation of raw milk cases occurred for a 6-year period, from 2012 to 2017 inclusive.

For the current analysis, anonymised data from the raw milk questionnaires previously collected were provided to the authors. Additional data for these cases, including case demographics and spatial location (meshblock), were obtained from the EpiSurv database. In addition, other cases of campylobacteriosis occurring in the area during the same period, with no reported raw milk consumption, were also obtained from EpiSurv to serve as a comparison population for analysis. This project was reviewed and approved by the MidCentral DHB ethics and Māori consultation boards, and has been recorded on the Massey University Ethics Low Risk Database.

### Description by person

#### Designation of cases

Raw milk campylobacteriosis cases, hereafter referred to as ‘raw milk cases’ were designated as those with reported raw milk exposure who, in addition to participating in a routine disease surveillance interview, had completed the specific raw milk questionnaire. A small number of additional cases who reported raw milk exposure in their interview, but who did not complete the raw milk questionnaire, were identified from the larger group of campylobacteriosis cases obtained via EpiSurv. Where sufficient data had been captured, these cases were also treated as raw milk cases. Campylobacteriosis cases who did not report raw milk consumption were deemed ‘non-raw milk cases’ and were used as a control population for parts of the analysis.

#### Demography of campylobacteriosis cases

Case data were summarised by counts and percentages for demographic variables. *χ*^2^ tests were used to compare case demographic variables by raw milk exposure status. Ethnicity was recoded into six groups using established protocols and then further collapsed into three groups; European, Māori and other, due to low numbers of other ethnicities [15]. Where respondents reported multiple ethnicities, output was reduced to one ethnicity following prioritised ethnicity guidelines [16]. Recorded urban/rural profile was reclassified from a seven-levelled variable into a binary variable and was thereafter coded as urban (previously classified as main urban area, independent urban area and satellite urban area) and rural (previously classified as highly rural/remote, rural with high urban influence, rural with moderate urban influence and rural with low urban influence).

A histogram of the distribution of age in years was examined visually by raw milk exposure and then recategorised into three age bands (0–9, 10–39 and ⩾40 years old) for *χ*^2^ analysis.

The proportions of raw milk cases by age group and rurality were estimated via logistic regression.

Occupation was reclassified using a modified version of the International Standard Industrial Classification (ISIC) [17]. Due to an interest in exposure to farm animals as a potential risk factor for disease, people who reported employment in meat handling or processing roles were placed into the agriculture, forestry and fishing group, rather than the group specified by ISIC – manufacturing. Due to low counts, occupation groups were collapsed from 17 to five by combining classes. A large number of cases were children and young adults in education and this was referenced by adding a class for children/students. A class was also added to include those not in paid employment – e.g. those described as retired, unemployed, receiving benefits or stay at home parents.

Cases had been classified as either outbreak-associated or sporadic (i.e. not recognised to be epidemiologically linked to an outbreak following investigation by the Public Health Unit). Hospitalisation status was used as a proxy for disease severity. *χ*^2^ analysis was performed to compare outbreak association and hospitalisation status by raw milk exposure. The Kruskal–Wallis test was used to compare the median ages of outbreak and non-outbreak cases.

#### Raw milk questionnaire

The raw milk questionnaire included a number of questions about factors that had motivated cases to consume raw milk. Cases were provided with some suggested motivations for raw milk consumption and, in response to their answer, these statements were marked ‘yes’ or ‘no’. Cases were also given the option to answer ‘other’ and have their responses captured in free text. In occasions where cases had indicated a view via free text that appeared to be clearly in support of one of the provided statements but had marked ‘no’ to the relevant question, this answer was changed to reflect the information contained in the free text.

In addition to the categories suggested by the questionnaire authors, a number of additional themes were obtained from the free text comments. To be defined as a theme, the topic needed to have been described by four or more respondents. Themes were generated independently by two researchers and then finalised by consensus. The degree of agreement between researchers was high, with both independently identifying the same four themes.

#### Sequence typing

Results from the campylobacter pathogen isolation and sequence typing of stool samples were available for the majority of cases. Sequence typing had been performed using the seven-gene multilocus sequence typing scheme described by Dingle *et al*. [12]. Where sequence type (ST) was not recorded, cases were retained as part of the dataset except in occasions where the sample was specifically noted as having been determined to not be campylobacter. The distribution of campylobacter multilocus STs was compared between raw milk and non-raw milk cases. Due to the presence of a large number of STs with low case counts, types that were associated with <3% of raw milk cases or non-raw milk cases were not examined in detail. Published source attribution data were used to examine the STs of raw milk cases and non-raw milk cases for trends towards poultry or ruminant strains.

### Disease trends over time

Campylobacteriosis cases were plotted over time to assess the temporal distribution of cases. Cases were aggregated by month and examined for temporal trends. The trends for raw milk cases were compared to non-raw milk cases. The seasonal pattern of cases was also assessed. For the purposes of this analysis, the seasons were defined as; summer – December to February, autumn – March to May, winter – June to August and spring – September to November.

#### Data analysis

All analyses were performed using RStudio (2016) with R version 3.4.4 (2018-03-15) – ‘Someone to Lean On’. Additional map generation was performed using QGis 2.14 ‘Essen’ (QGIS Development Team (2017)).

## Results

### Description by person

#### Designation of cases

The number of notified cases of campylobacteriosis that were reported in the MidCentral DHB area between 2012 and 2017 was 1408. Campylobacter sequence typing did not support a diagnosis of campylobacteriosis in eight cases and these were excluded from the analysis. Ninety-three cases reported raw milk consumption and completed the raw milk questionnaire. An additional 12 cases, who reported raw milk consumption but did not complete the raw milk questionnaire, were identified from amongst the remaining campylobacteriosis cases using surveillance data from EpiSurv. These additional cases were also deemed raw milk cases for the purposes of demographic analysis.

#### Demographics of campylobacteriosis cases

**Fig. 2.**
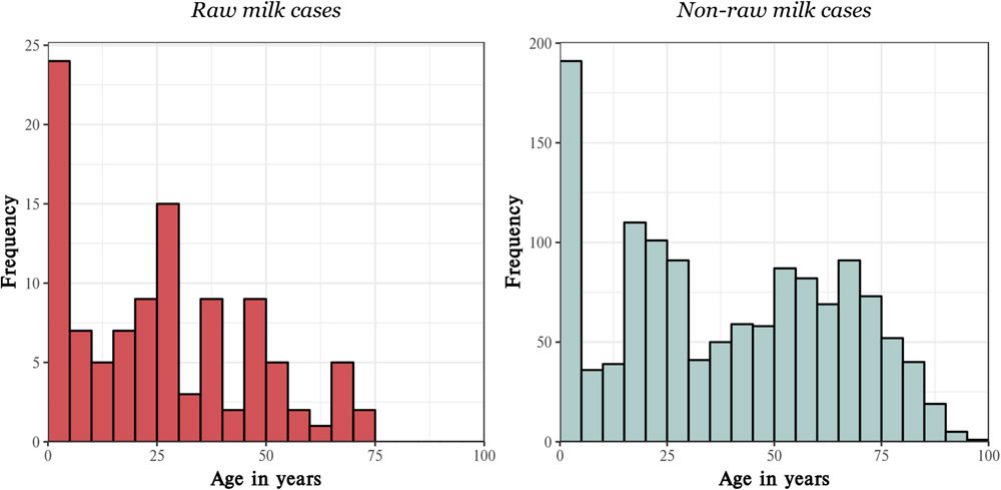
Age distribution of raw milk and non-raw milk exposed campylobacteriosis cases from the area serviced by the MidCentral DHB, 2012–2017.

Age was non-normally distributed. The median age was 26 (range 0–75) for raw milk cases and 39 (range 0–97) for non-raw milk cases. A peak in cases amongst children aged 5 years old or less was observed for both groups. A second peak in cases was apparent for raw milk cases aged in their early to mid-20s. The comparative age distributions for both groups are shown in Figure 2.

Demographic variables, including age reclassified into three groups, are summarised for raw milk and non-raw milk cases in Table 1. Raw milk cases were roughly evenly split by gender. A larger percentage of non-raw milk cases were male; however, proportions were not significantly different on *χ*^2^ analysis when compared to raw milk cases. Percentages by ethnic group were similar between exposure groups and were not statistically different on *χ*^2^ analysis. A larger percentage of raw milk than non-raw milk cases resided in a rural area; however, this difference was not significant (*P* = 0.27). The *χ*^2^ test results were highly significant (*P* < 0.001) for age by raw milk exposure status. A greater percentage of non-raw milk cases were aged 40 years and older compared to raw milk cases, whereas a proportionately larger percentage of raw milk cases were <10 years old. There was a higher proportion of raw milk cases amongst 0–9 years old in rural areas compared to amongst urban cases in this age group; however, proportions were not significantly different. The proportions of raw milk cases over total campylobacter cases, stratified by age group and rurality, are provided in Table 2.

**Table 1.** Demographics of 1400 notified campylobacteriosis cases, stratified by raw milk exposure, from the MidCentral DHB area, 2012–2017

Variable	Level	Raw milk cases *n* (%)	Non-raw milk cases *n* (%)	*P*-value^a^
Gender				0.23
	Female	52 (49.5)	558 (43)	
	Male	53 (50.5)	737 (57)	
	Total	105	1295	
Ethnicity				0.52
	European	86 (82.7)	1057 (84.2)	
	Māori	14 (13.5)	130 (10.4)	
	Other	4 (3.8)	67 (5.3)	
	Total	104	1254	
Urban/rural profile				0.27
	Rural	32 (33)	336 (27.0)	
	Urban	66 (67)	908 (73.0)	
	Total	98	1244	
Age (years)				<0.001
	0–9	30 (28.6)	220 (16.9)	
	10–39	46 (43.8)	431 (33.3)	
	⩾40	29 (27.6)	644 (49.7)	
	Total	105	1295	

^a^Statistical significance assessed via *χ*^2^ analysis.

**Table 2.** Counts and proportions of raw milk cases (over total cases) by age group, stratified by urban/rural status

Urban/rural profile	0–9 years^a^	10–39 years	⩾40 years
Rural	17/99 (0.17) [0.11–0.26]	10/120 (0.08) [0.05–0.15]	5/149 (0.03) [0.01–0.08]
Urban	11/141 (0.08) [0.04–0.14]	32/338 (0.09) [0.07–0.13]	23/493 (0.05) [0.03–0.07]

^a^Values reported are counts and proportions of raw milk cases over total cases and 95% confidence intervals of proportions as determined by logistic regression.

A summary of cases by occupation is presented in Table 3. Results of *χ*^2^ tests indicated that there were significant differences between raw milk and non-raw milk cases by occupation group (*P* = 0.01). Around half of all campylobacteriosis cases were in education or not employed. A greater proportion of raw milk cases were children or students compared to non-raw milk cases. Conversely, proportionately more non-raw milk cases were unemployed or not in work. For other occupation classes, numbers were relatively similar between exposures. The percentages of cases employed in agriculture, forestry and fishing were similar between raw milk and non-raw milk cases.

**Table 3.** Occupation group of 1400 notified campylobacteriosis cases from the MidCentral DHB region, 2012–2017, stratified by raw milk exposure

Occupation Group	Raw milk cases *n* (%)	Non-raw milk cases *n* (%)	*P*-value^a^
Agriculture, forestry, fishing	14 (13.9)	131 (11.1)	0.01
Administration and support	5 (5.0)	74 (6.3)	
Construction and manufacturing	8 (7.9)	55 (4.7)	
Profession, scientific, technical	5 (5.0)	81 (6.9)	
Public administration, education and health	5 (5.0)	101 (8.6)	
Children and students	40 (39.6)	315 (26.7)	
Retired/unemployed/stay at home parent	12 (11.9)	309 (26.2)	
Other	12 (11.9)	112 (9.5)	
Total	101	1178	

^a^Statistical significance assessed via *χ*^2^ analysis.

#### Disease severity and outbreak association

Twelve raw milk cases and 33 non-raw milk cases were recorded as being associated with an outbreak. Outbreak status was not recorded for 35 non-raw milk cases. The median number of cases per outbreak was three for raw milk cases (range 2–4) and two for non-raw milk cases (range 1–4). The percentage of outbreak cases was significantly greater for raw milk compared to non-raw milk cases (*P* < 0.001). Around 13% of all campylobacteriosis cases were hospitalised, with no difference seen between exposures. The median age of both raw milk and non-raw milk-associated outbreak cases was 30. However, there was greater variability in age for non-raw milk cases (range 1–95, IQR 51), compared to raw milk cases (range 1–52, IQR 35). There was no significant difference between the median age of the groups when assessed by Kruskal–Wallis test (*P* = 0.35). The outbreak and hospitalisation status of cases is summarised in Table 4.

**Table 4.** Outbreak association and hospitalisation status for 1400 notified campylobacteriosis cases from the MidCentral DHB region, 2012–2017, stratified by raw milk exposure

Variable	Level	Raw milk cases *n* (%)	Non-raw milk cases *n* (%)	*P*-value^a^
Outbreak-associated				
	Yes	12 (11)	33 (2.6)	<0.001
	No	93 (89)	1260 (97.5)	
	Total	105	1293	
Hospitalised				
	Yes	14 (13)	159 (13.5)	1
	No	91 (87)	1021 (86.5)	
	Total	105	1180	

^a^Statistical significance assessed via *χ*^2^ analysis.

### Raw milk questionnaire

#### Motivations for raw milk consumption

Results from 93 raw milk questionnaires were available. Most cases had not answered all questions in the questionnaire. Cases did not appear to consistently support any of the five reasons for consumption suggested in the questionnaire; however, 59 cases (63%) answered ‘yes’ to at least one question. ‘Health reasons’ appeared to be the category with the most support (31% agreed). ‘Support for local producers’ did not appear to be a driver of consumption with only 2% of cases agreeing. Thirteen responses were changed to match the comments provided in the free text. A summary of responses to postulated motivators of raw milk consumption is provided in Table 5.

**Table 5.** Responses to researcher-proposed motivations for raw milk consumption in notified campylobacteriosis cases – MidCentral DHB region, 2012–2017 (*n* = 94)

Variable	Level	Frequency	Percentage
Health reasons			
	Yes	29	31
	No	48	51
	Non-response	17	18
Natural			
	Yes	17	18
	No	54	57
	Non-response	23	25
Produced on farm			
	Yes	21	22
	No	50	53
	Non-response	21	22
Cheap			
	Yes	17	18
	No	59	63
	Non-response	18	19
Support local producers			
	Yes	2	2
	No	69	73
	Non-response	22	23

Other common themes motivating raw milk consumption that were identified via free text from the raw milk interviews included taste, convenience, product received as a gift and a desire to try raw milk. Of these, taste and convenience were the most frequently reported (20 and 12 respondents, respectively). Product received as a gift and the desire to try raw milk were both reported by four cases each.

#### Sequence typing

One hundred and nine distinct campylobacter multilocus STs were identified from notified campylobacteriosis cases during the study period, with 29 individual STs reported in raw milk cases and 105 in non-raw milk cases. Fifty-one of the STs were each isolated from only one case, respectively. Of 105 raw milk cases and 1295 non-raw milk cases, STs were not available for 30 raw milk cases and 462 non-raw milk cases.

The most common ST overall was ST 45 (117 cases). Of the 117 cases from which ST 45 was isolated, 116 were non-raw milk exposed, making this also the most common non-raw milk isolate (13.9% of sequenced non-raw milk cases). STs 50 and 53 were also frequent isolates seen in non-raw milk cases, making up 7.3% and 6.2% of sequenced non-raw milk cases, respectively.

**Fig. 3.**
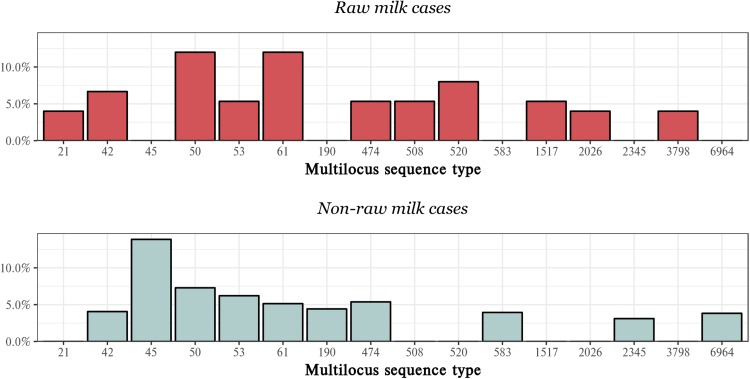
Percentage of notified campylobacteriosis cases by multilocus sequence type, stratified by raw milk exposure – MidCentral DHB region, 2012–2017. STs comprising <3% of cases respectively have been excluded except where the ST was reported frequently in the opposing exposure group.

The most frequently reported raw milk STs were 50 and 61 which were each isolated from 12% of raw milk cases. STs 520 (8% of sequenced raw milk cases) and 42 (7% of sequenced raw milk cases) were also important raw milk isolates. Five STs were commonly isolated from both raw milk and non-raw milk cases. A summary of the most frequently reported STs by raw milk and non-raw milk exposure is presented in Figure 3.

### Disease trends over time

#### Notified campylobacteriosis cases from MidCentral DHB 2012–2017

**Fig. 4.**
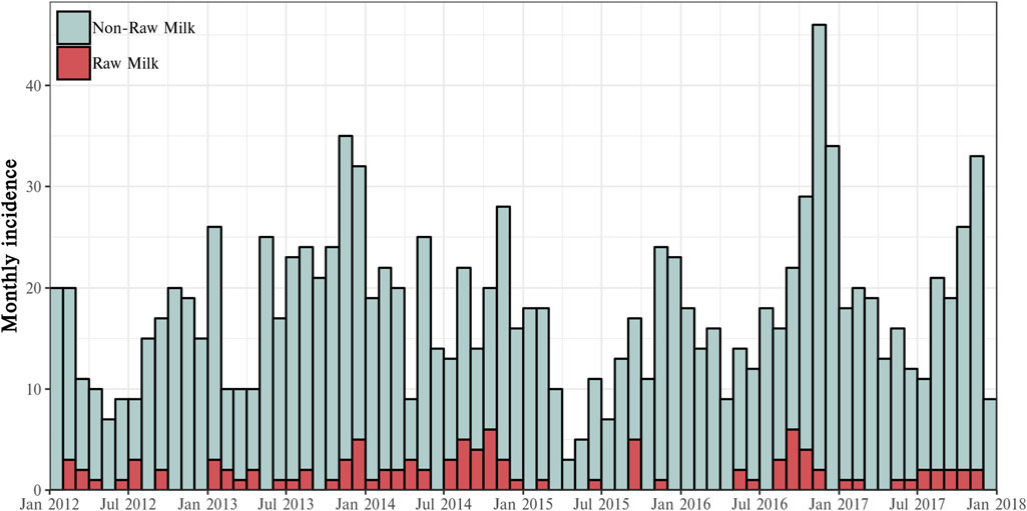
Notified campylobacteriosis cases for the MidCentral DHB region by raw milk exposure, 2012–2017.

The number of notified cases of campylobacteriosis reported in the MidCentral DHB area between January 2012 and December 2017 was 1400. The largest number of campylobacteriosis cases by month was 46, occurring in November 2016. Smaller peaks also occurred in November/December 2013 and December 2016. Raw milk cases made up between 0% and 33% of campylobacteriosis cases on a per month basis. The distribution of notified campylobacteriosis cases by month, stratified by raw milk exposure, is presented in Figure 4.

#### Seasonal patterns of disease

**Fig. 5.**
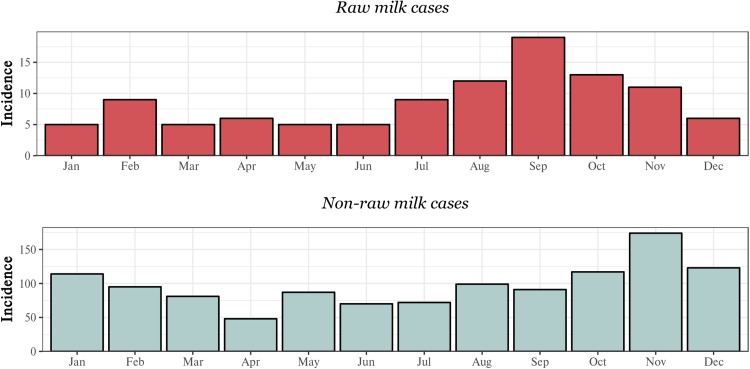
Monthly distribution of campylobacteriosis cases, by raw milk exposure, for the MidCentral DHB, 2012–2017.

A summary of raw milk and non-raw milk cases, aggregated by month, is presented in Figure 5. Raw milk case numbers were proportionately lower during the summer, autumn and early winter but showed evidence of a seasonal peak that began in late winter and continued through spring. The greatest number of cases occurred in September – 19 of 105 (18%) of cases. The number of raw milk cases dropped noticeably in December as summer began.

In contrast, non-raw milk case numbers appeared more evenly spread throughout the year but were at their lowest during autumn and winter. A spring peak was also evident for non-raw milk cases, but occurred later in this group, with peak incidence arising in November. Unlike raw milk cases, the peak in non-raw milk cases did not appear to be confined to spring, continuing into the summer months.

## Discussion

This study examined the demographics of notified campylobacteriosis cases with reported raw milk exposure from a sentinel surveillance site in New Zealand and compared them with unexposed campylobacteriosis cases from the same period. Analysis found that cases of raw milk-associated campylobacteriosis were more frequently reported in younger children compared to non-raw milk campylobacteriosis cases. These results were consistent with previous investigations that concluded that children tend to be disproportionately affected by raw milk-associated disease [18–20]. Elderly individuals have also been found to be more susceptible to milk-borne pathogens [19]; however, this finding was not supported by the results of this study. It is possible that raw milk exposure could be a proxy for other risk factors, especially among younger cases. Rural children who live on farms will have a greater chance of contact with livestock or other animals and may also be exposed to untreated drinking water. It could also be hypothesised that raw milk consumers might also practice other food consumption habits that could increase the risk of disease. One study found that the proportion of raw milk drinkers who reported consuming other risky food items, such as raw oysters and unpasteurised juice, was higher than that of pasteurised milk consumers [18].

It is difficult to determine from the existing data whether age-related differences in raw milk-associated campylobacteriosis rates were due to variations in susceptibility between groups or to differences in consumption rates between people of different ages. Comprehensive information about the raw milk drinking habits of New Zealand residents is not available, limiting conclusions that can be drawn. An American cross-sectional telephone study, however, found that individuals who consumed raw milk had similar gender and age demographics to pasteurised milk consumers [18]. UK and US data suggest that children aged <11 years consume more milk than older children and adults [21,22]. A New Zealand national nutrition survey did not quantify the amount of product consumed but found that the proportion of people drinking milk seven or more times a week decreased significantly with age [23]. If raw milk consumption patterns are similar to those reported for pasteurised milk, it cannot be ruled out that the increased rate of raw milk disease seen in children may be due in part to greater exposure rather than greater susceptibility.

Occupation by group was significantly different between raw milk and non-raw milk cases (*P* = 0.01). However, examination of the data suggested that observed differences arose primarily due to different proportions of children and students and retired/unemployed people between the two exposure groups. This likely reflected the difference in age structure between the two exposure groups noted previously. Proportions of cases working in agriculture, forestry and fishing were similar between groups. Occupational contact with livestock is a known risk factor for campylobacteriosis in general [24], however, to the authors' knowledge, the association between the risk of raw milk-related disease and agricultural occupation has not been systematically investigated. If campylobacteriosis is more common in people with agricultural exposures, the choice of other campylobacteriosis cases as a control group for raw milk cases may have resulted in a selection bias that has the potential to obscure risk factors that are more common in both groups.

Proportions of raw milk and non-raw milk cases by gender and ethnicity were not found to be statistically different. A greater percentage of raw milk cases were living in a rural location compared to non-raw milk cases (33% and 27%, respectively); however, this difference was not significant (*P* = 0.27). The consumption of raw milk is known to be more common amongst dairy farming households than other New Zealand households [7]; however, the present study does not show an overall association between rurality and raw milk-associated campylobacteriosis compared to other types of campylobacteriosis. Study data suggested that the proportion of raw milk cases was higher in rural 0–9 years old, compared to urban children of the same age. However, there was not sufficient data to show this statistically due to relatively small counts within this group (17 and 11 cases, respectively). The relatively low numbers of raw milk disease cases in this study may have been a limiting factor in this analysis.

A significantly greater percentage of raw milk cases were associated with a recognised outbreak compared to non-raw milk cases (*P* *<* 0.001). This is consistent with previous studies that have found dairy products to be the most common vehicle associated with food-borne disease outbreaks of *Campylobacter jejuni*, in contrast to poultry-related campylobacteriosis which is more likely to cause sporadic disease [25–27]. Limited literature is available allowing the comparison of the proportions of outbreak *vs.* sporadic raw milk-associated cases. However, a review of foodborne disease surveillance in Minnesota, USA also found that it was likely that substantially more raw milk-associated cases occurred sporadically rather than in association with a recognised raw milk-associated outbreak [28].

None of the putative motivations for raw milk consumption suggested by researchers appeared to resonate widely with raw milk cases in the present study. The suggested motivation with the greatest support was ‘health reasons’, with 31% in agreement. It has previously been found that many raw milk consumers believe that raw milk consumption has health benefits [19,29]. In a Ministry for Primary Industries (MPI) anonymous survey examining New Zealanders' experiences of buying and consuming raw milk, 92% of the raw milk consumers surveyed stated that they consumed raw milk due to ‘health benefits’, a higher percentage than in the present study [30]. Taste was not a pre-specified category in the current study but appeared to be a relatively popular reason given in free text answers (20 cases in agreement). Previous international studies have also found taste to be an important driver of demand for raw milk [19,29] and New Zealand consumers concurred with this in the MPI survey, with 96% in agreement [30]. While previous international studies found that support for local farmers, a desire for more natural products and a belief that raw milk-producing dairy farms were more humane in their animal management were important motivators for raw milk consumption, respondents to the current study did not appear to find these significant drivers [29,31,32]. It should be noted that the participants in the present study had a different selection criteria compared to those enrolled in previous surveys and this may explain some of the differences in responses described. It is unknown to what extent development of an episode of disease may retrospectively affect a person's view of the motivations that previously prompted them to consume raw milk.

Sequence typing of raw milk and non-raw milk campylobacteriosis cases revealed that a range of STs were associated with disease. Some STs were more often associated with raw milk cases than non-raw milk; however, there was an overlap between exposures. The majority of frequently reported STs from raw milk cases have been previously shown to be more likely to be isolated from ruminants than other sources [33,34]. The exception was ST 474 which is considered to be more frequently poultry-associated [34]. It is acknowledged that, as the focus of the current analysis was on raw milk-associated disease, cases that had reported raw milk exposure have been designated raw milk cases without consideration of alternative risk factors that may represent other potential sources of disease for these individuals.

A seasonal peak in raw milk cases, occurring in late winter and into the spring, was apparent from the examination of the data. An obvious putative cause of this peak is the seasonal nature of milk supply in New Zealand, with the majority of herds beginning calving in July and continuing into September. The start of the lactation period is likely to represent the return, or increase in availability, of raw milk to consumers. People who live on farms and consume raw milk produced there may also swap to pasteurised milk during the dry period for their herd. Repeated exposure to campylobacter organisms has been postulated to provide immunity sufficient to prevent severe clinical illness [35]. Limited data from challenge studies exist to enable the establishment of the duration of immunity following exposure; however, it has been shown that complete protective immunity may wane over a period of months [36]. The resumption of raw milk consumption for consumers, following a period of reduced exposure over winter, may therefore be associated with a greater risk of disease. In the case of farm-based raw milk consumers, it is noted, however, that during the calving period, this group may also have a greater risk of contracting campylobacteriosis via other pathways, due to necessary increases in contact with animals during this time. This has the potential to confound the relationship between a suspected increase in raw milk consumption during spring and the observed spring peak in cases for these consumers.

In addition to a likely increase in exposure to raw milk, the higher numbers of campylobacteriosis cases seen in the spring months may also be due to seasonal variations in the prevalence of campylobacter within the faeces of dairy cattle. In a UK study, Stanley *et al*. found distinctly greater numbers of campylobacter bacteria in the fresh faeces of dairy cattle during spring and autumn, with consistent cyclical patterns seen over a number of years [37]. These authors noted that this bimodal pattern did not directly match the observed single spring peak in human campylobacteriosis cases seen in the UK but did roughly coincide with spring and autumn increases reported in the USA [37]. In contrast, non-raw milk campylobacteriosis has traditionally been associated with summer peaks in New Zealand, which are considered largely attributable to poultry sources [24]. This distribution was apparent in the current study, with a later and slightly more diffuse peak seen in non-raw milk cases.

Some limitations exist in the current study. First, the sample size was relatively small, particularly with regards to raw milk case numbers. Second, the data available were not complete for every case. Complete data were particularly desirable for raw milk cases which were the focus of the review. It has been reported that some raw milk cases may be reluctant to answer questions about the subject [38]. In the current analysis, it is not known why 12 cases with raw milk exposure did not complete the raw milk questionnaire. There is also the potential for cases with raw milk exposure to deny consumption altogether, which may contribute to an underestimation of the number of cases associated with this exposure [28]. Alternatively, as most cases were sporadic, making it more difficult to definitively link disease to raw milk exposure, there is the potential that disease may have been incorrectly attributed to raw milk consumption in the presence of other risk factors. As discussed previously, some raw milk cases had other reported exposures which may have been the cause of their disease.

Despite the potential for some misclassification of raw milk cases, it is considered more likely that the current study underestimated rather than overestimated the overall numbers of raw milk-associated campylobacteriosis cases in the region [39,40]. It is also noted that the current study examined only campylobacteriosis cases and excluded other raw milk pathogens, some of which, while less commonly notified, are associated with a greater risk of severe disease [4].

The choice of the MidCentral DHB as the study region largely arose due to the region's role as a sentinel site for campylobacteriosis investigation and the extension of this research into an analysis of raw milk-associated disease. The representativeness of this region compared to greater New Zealand has been previously assessed [12]. It was concluded that while the region was representative of national variability in many measures, its demographics did vary from the population of New Zealand as a whole, with differences seen in age structure, urban/rural profile and ethnicity compared to other regions [12]. On the other hand, the enhanced data captured in the MidCentral sentinel site lent itself well to a pilot analysis of raw milk disease, as the data for this DHB were considered more complete for this research area [24].

## Conclusion

The results of this analysis indicated that rural children appeared to be at an increased risk of raw milk-associated campylobacteriosis compared to non-raw milk-associated disease. It is unknown whether this association was due to increased exposure to raw milk, increased susceptibility among this group or a combination of these factors. Stakeholder engagement with rural networks such as Federated Farmers and Dairy Women's Network should be enhanced to co-design appropriate public health messaging to target this group.

A seasonal pattern was apparent in raw milk-associated campylobacteriosis, with a peak in cases seen in the spring. This rise in cases may be due to both increased access to raw milk during this period and observed seasonal increases in the shedding of campylobacter organisms in dairy cattle.

Finally, the motivations of raw milk cases in the current study did not appear to match those proposed by the raw milk questionnaire authors. It is suggested that this area may be important to target in order to develop an effective public health response to raw milk-associated disease, as some consumer motivations may be able to be satisfied by the development of alternative milk products that meet consumer demands in a safer manner than raw milk.
